# Synthesis of New Bis(3-hydroxy-4-pyridinone) Ligands as Chelating Agents for Uranyl Complexation

**DOI:** 10.3390/molecules21030299

**Published:** 2016-03-08

**Authors:** Bo Jin, Rongzong Zheng, Rufang Peng, Shijin Chu

**Affiliations:** State Key Laboratory Cultivation Base for Nonmetal Composites and Functional Materials, Southwest University of Science and Technology, Mianyang 621010, China; 6070831@sina.com (R.Z.); chushijin@swust.edu.cn (S.C.)

**Keywords:** chelating agent, 3-hydroxy-4-pyridinone, uranium, tetradentate chelator

## Abstract

Five new bis(3-hydroxy-4-pyridinone) tetradentate chelators were synthesized in this study. The structures of these tetradentate chelators were characterized by ^1^H-NMR, ^13^C-NMR, FT-IR, UV-vis, and mass spectral analyses. The binding abilities of these tetradentate chelators for uranyl ion at pH 7.4 were also determined by UV spectrophotometry in aqueous media. Results showed that the efficiencies of these chelating agents are dependent on the linker length. Ligand **4b** is the best chelator and suitable for further studies.

## 1. Introduction

Uranium is introduced into the body by ingestion, inhalation, or through wounds. The risk of uranium contamination has considerably magnified because of the extensive use of uranium as nuclear fuel in fission reactors and as weapon-grade nuclear material. The hexavalent uranyl ion [UO_2_^2+^, U(VI)] is the most stable form *in vivo* [1] and is complexed in the blood by chelating agents, such as proteins or carbonates. Meanwhile, tissues, especially the kidney and bones, accumulate uranium for months to years, which will induce cancer and chemical intoxication [2,3,4]. Thus, uranium should be eliminated from the body by administration of nontoxic chelating agents that can form stable complexes with the uranyl ion.

Among the different chelators, 3-Hydroxy-4-pyridinones (3,4-HOPO) have emerged as one of the hotspots in studies that focus on heavy metal chelators because of their special bidentate structure, highly selective chelating capacity, and significant physiological activities [5,6,7,8,9,10,11,12]. To date, three kinds of 3,4-HOPO derivatives are available, namely bidentate hydroxypyridinones [5,6], tetradentate hydroxypyridinones [7,8,9], and hexadentate hydroxypyridinones [10,11]. The ideal design for chelators is to synthesize the hydroxypyridinones, which have excellent chelating efficacy and high selectivity of interaction, with special biological receptors in one molecular unity. The chelating capacity of bidentate hydroxypyridinones is usually inferior to that of hexadentate desferrioxamine [13,14]. Hexadentate hydroxypyridinones have a higher chelating ability than hexadentate desferrioxamine, but the poor absorption caused by their high molecular weight limits their application [15]. Thus, tetradentate hydroxypyridinones have been one of most extensively investigated compounds among heavy metal chelators [16]. Recent studies have reported that the tetradentate hydroxypyridinones exhibit better assays *in vivo* such as high chelating efficacy for Fe and Ga as well as excellent hydrophilic character [17,18,19,20]. However, few studies have examined the hydroxypyridinone chelating uranyl ions. In this paper, a series of new tetradentate hydroxypyridinone chelators is reported, and the binding affinities towards a uranyl cation (UO_2_^2+^) were examined with UV spectrophotometry.

## 2. Results and Discussion

### 2.1. Synthesis

**Scheme 1:**
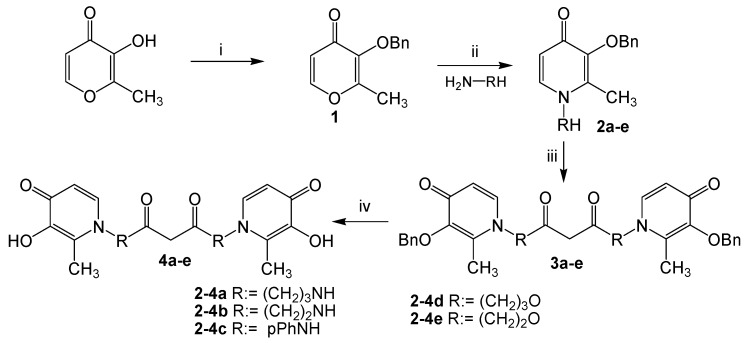
Synthesis for the ligands **4a**–**e**. *Reagents and conditions*: (i) BnBr, CH_3_OH, 75 °C, reflux 6 h; (ii) EtOH/H_2_O, NaOH, reflux 3 h, 10 M HCl, until pH = 1, 10 M NaOH, pH = 11; (iii) CH_2_(COCl)_2_, dried CH_2_Cl_2_, 0 °C, 8 h; (iv) H_2_, Pd/C, CH_3_OH, 4 h.

The synthesis of tetradentate hydroxypyridinones **4a**–**e** is shown in Scheme 1. Starting from the commercially available 3-hydroxy-2-methyl-4-pyrone (maltol), the hydroxyl was protected with benzyl bromide and proceeded in excellent yield (93%) to produce 3-benzyloxy-2-methyl-4-pyrone **1**. Then 3-Benzyloxy-2-methyl-4-pyrone **1** was directly condensed with diamines or ammonia alcohols to provide benzyloxy-pyridone derivatives **2a**–**e** in 75%–89% yields. This method was similar to that described by Santos [18] but with minor modifications. In this study, for benzyloxy-pyridone **2a**, compared with the method of Santos, the molar ratio of amines with pyrone **1** was increased from 1:1 to 3:1, and the corresponding yield of **2a** evidently increased from 40% to 87%. The structures of benzyloxy-pyridones **2a**–**e** contained a reactive hydroxyl or amino group, which could easily react with malonyl dichloride to yield the corresponding condensed products **3a**–**e**. The malonyl dichloride is extremely active, so the reaction should be conducted in an ice bath and anhydrous conditions. Removal of benzyl from the oxygen of **3a**–**e** catalytic hydrogenation conditions (14.5 psi H_2_, 10% Pd/C as catalyst) proceeded smoothly with good yield (71%–90%) to produce tetradentate hydroxypyridinones **4a**–**e**.

### 2.2. Characterization

All products were purified and characterized by FT-IR, NMR, UV-vis and MS, and all characterizations were in accordance with the structures of the products.

**Figure 1:**
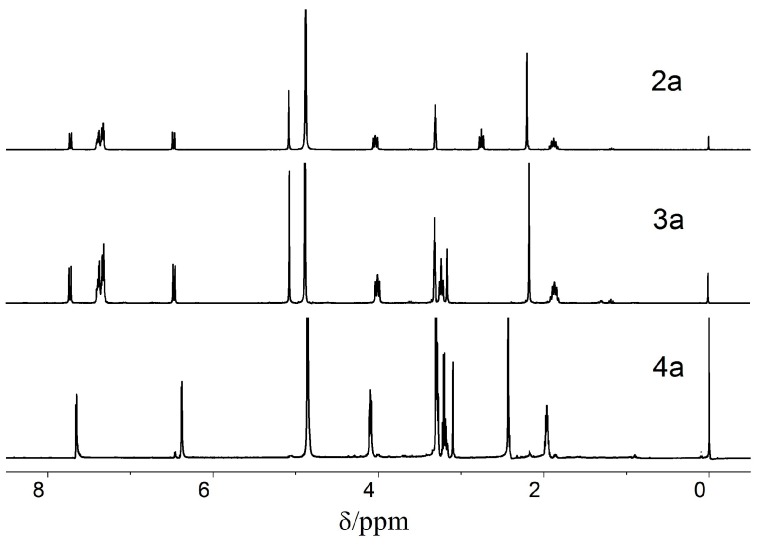
The ^1^H-NMR spectra of **2a** (CD_3_OD), **3a** (CD_3_OD) and **4a** (DMSO-*d*_6_).

The ^1^H-NMR spectra of compounds **2**–**4a** are shown in Figure 1. Compared with **2a**, the signal of NHC*H_2_*CH_2_- in **3a** shifted to the low chemical field (from 2.75 to 3.31) because of the electrophilic effect of the –CONH, and the singlet of the –COC*H*_2_CO– in **3a** appeared at δ = 3.20 ppm. Compound **4a** was obtained after the hydroxyls of **3a** were deprotected. The peaks at 7.34 ppm of the benzene ring and 5.07 ppm of the methylene disappeared when the benzyl group was removed, and the signal of the *CH*_3_-Pys shifted from 2.16 ppm to 2.42 ppm.

The ^1^H-NMR spectra of **2b**–**e**, **3b**–**e**, and **4b**–**e** have similar results. The ^1^H-NMR spectra of **4b** and 4**c** exhibit two doublets at 7.6 ppm–7.7 ppm and 6.45 ppm–6.55 ppm with *J*_AB_ = 7.5 Hz for the two nonequivalent protons in the pyridine ring. Compared with **4d** and **4e**, the signals of the pyridine ring protons appeared in the higher field because the oxygen atom is more electronegative than the nitrogen atom. The signals of the two nonequivalent protons in the pyridine rings of **4d** and 4**e** appeared at 8.30 ppm–8.33 ppm (d, *J* = 7.2 Hz, 1H) and 7.23 ppm–7.25 ppm (d, *J* = 7.2 Hz, 1H), respectively. Additionally, the signals of –COC*H_2_*CO– in **4d** and **4e** appeared in a lower field than in **4b** and **4c** under the same condition. The singlet of –COC*H*_2_CO– in **4b** and **4c** appeared at 3.0 ppm–3.1 ppm, whereas that of **4d** and **4e** appeared at 3.6 ppm–3.8 ppm, respectively.

### 2.3. Complexation

Although present metal-complexation studies are focused on a set of three-charged hard metal ions [21,22] (e.g., Fe, Al, and Ga), the current study on decorporation [23] for UO_2_^2+^ is based on the extreme damage to the environment caused by uranyl cations (UO_2_^2+^) in biological systems. In this paper, the complexation behavior of tetradentate hydroxypyridinones **4a**–**e** and the uranyl cation was evaluated by the spectrophotometric method [24,25].

We defined:

M = UO_2_^2+^; L = ligands **4a**–**e**

C = [M] + [L]; x = [M]/C

**Figure 2:**
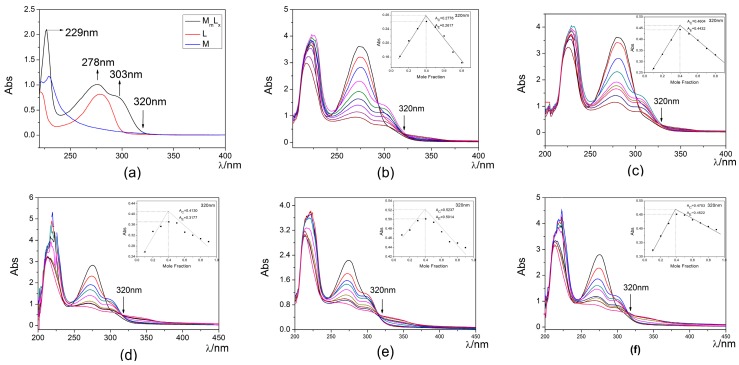
UV-vis absorption spectra of the various metal-to-ligand molar ratios for the UO_2_^2+^-Ligand **4a**–**e** system at pH 7.4: C = 4.00 × 10^−4^ M, T = 25 °C, I = 0.1 M KNO_3_: (**a**) The spectrophotometric absorption curves of the UO_2_^2+^, ligand **4a** and UO_2_^2+^-ligand **4a**; (**b**) UO_2_^2+^-Ligand **4a** system; (**c**) UO_2_^2+^-Ligand **4b** system; (**d**) UO_2_^2+^-Ligand **4c** system; (**e**) UO_2_^2+^-Ligand **4d** system; (**f**) UO_2_^2+^-Ligand **4e** system. All illustrations were obtained from the wavelength of 320 nm.

The coordination numbers (M:L) and the corresponding complex stability constants of the five tetradentate hydroxypyridinones **4a**–**e** toward UO_2_^2+^ at pH 7.4 were measured with the method of equivalent molarity. The UV-vis spectra of the various metal-to-ligand molar ratios for the UO_2_^2+^-ligand **4a**–**e** system at pH 7.4 are shown in Figure 2. UO_2_^2+^ has an absorption peak at 229 nm, the peak at 278 nm was a characteristic absorption of ligand **4a**, and a new peak at 303 nm appeared, which indicated that a new peak was the characteristic of the UO_2_^2+^-ligand **4a** complexation (Figure 2a). To construct the Job plot, the absorbance values were normalized according to the complex absorbance, and the experimental absorbance values of the UO_2_^2+^-ligand **4a** complexation at pH 7.4 are plotted against the mole fraction (Figure 2b). The result shows that the complex stoichiometry for the complexation of UO_2_^2+^-ligand **4a** at pH 7.4 is *M*/*L* = 2:3 (x = 0.4). This value indicates that the three ligand molecules could bridge two bis-chelated UO_2_^2+^ centers under the effect of polar solvent molecules to complete their coordination sphere at pH 7.4.

Then ligands **4a**–**e** complex with the uranyl, and the formation constants (log K) for the ligands could be calculated as follows:

**2 M****+****3 L****=****M_2_L_3_**Initial concentration:xC
(1 − x)C
0Equilibrium concentration:[M]_E_
[L]_E_
[M_2_L_3_]_E_Complete reaction:0
0
[M_2_L_3_]_C_

Based on the Lambert-Beer law:

$$\frac{A_{E}}{{\left[M_{2}L_{3}\right]}_{E}}=\frac{A_{C}}{{\left[M_{2}L_{3}\right]}_{C}}\Rightarrow {\left[M_{2}L_{3}\right]}_{E}=\frac{A_{E}{\left[M_{2}L_{3}\right]}_{C}}{A_{C}}$$

(1) $$\log K=\frac{{\left[M_{2}L_{3}\right]}_{E}}{{\left[M\right]}_{E}^{2}{\left[L\right]}_{E}^{3}}=\log\frac{{\left[M_{2}L_{3}\right]}_{E}}{{\left\{xC-2{\left[M_{2}L_{3}\right]}_{E}\right\}}^{2}{\left\{(1-x)C-3{\left[M_{2}L_{3}\right]}_{E}\right\}}^{3}}$$

The complex stability constant of **4a** and UO_2_^2+^ at pH 7.4 was calculated by Equation (1), and the corresponding logK_cond_U-L **4a** at 7.4 pH levels was 21.7. The corresponding complex stability constants of other ligands were also calculated by Equation (1), and the results are shown in Table 1.

Although the structures of Ligands **4a**–**e** are similar, some consistent differences are apparent. The change of the linker length has a great influence on the their U(VI) chelation efficiency [26,27,28]. As the length of the linker increases, the angle formed between the uranium and two phenolic oxygen donors also becomes greater, and it leads to an increase of the strain in this complex. [29] As a result of the strain imposed by the linker, that two carbon atoms may be considered the optimal length is consistent with the high efficacy of ligands **4b** and **4e** for in vivo uranyl chelation. Ligands **4b** and **4e** exhibit the highest K_cond_ at pH 7.4, and the corresponding logK_cond_ are 22.7 and 22.2. The modest reduction of body uranium in animals by the injection of bidentate Tiron (4,5-dihydroxy-1,3-benzenedisulfonic acid, disodium salt) and its U(VI)-catechol complex (log KML ) is 15.9 [29,30,31], which suggests the U(VI)-Ligands **4b** complex and U(VI)-Ligands **4e** complex Ligands 4b and 4e exhibit higher stability than the U(VI)-Tiron complex. Thus, Ligands **4b** and **4e** are most suitable for further studies.

## 3. Materials and Methods

### 3.1. General

The organic reagents used were pure commercial products from Aladdin. The solvents were purchased from Chengdu Kelong Chemical Reagents Co. (Sichuan, China). Anhydrous CH_2_Cl_2_ was distilled prior to use. The 300–400 mesh silica gel was purchased from Qingdao Hailang. The ^1^H-NMR, ^13^C-NMR spectra were recorded on Bruker Avance 300, Avance 400, or Avance 600 spectrometer (Carlsruhe, Germany). The FTIR spectra were obtained from Nicolet 380 FTIR spectrophotometer (Thermo Fisher Nicolet, Madison, WI, USA) with a resolution of 4 cm^−1^ from 400 cm^−1^ to 4000 cm^−1^. UV-vis spectrophotometer (Thermo Scientific Evolution 201, Waltham, MA, USA) used had a double-beam light source from 190 nm to 1100 nm. Mass spectral analysis was conducted using Varian 1200 LC/MS (Palo Alto, CA, USA).

### 3.2. Synthesis

*Synthesis of 3-benzyloxy-2-methyl-4-pyrone* (**1**). To a solution of 10.00 g 3-hydroxy-2-methyl-4-pyrone (79 mmol) containing the equivalent amount of NaOH (3.16 g, 79 mmol) in 100 mL methanol, 13 mL benzyl bromide (90 mmol) was dropwise added and then stirred for 6 h under reflux temperature. After cooling, the reaction mixture was evaporated under vacuum, and the residual oil was resolved in 50 mL dichloromethane and washed with 5% NaOH aqueous solution (5 × 30 mL) and water (3 × 50 mL). The organic solution was evaporated to dryness to obtain the pure product as pale oil (15.7 g, 93%). UV-vis (CH_3_OH): λ_max_ = 219, 260 nm. FT-IR (KBr): ν = 3065, 3031, 2958, 2877, 1644, 1575, 1496, 1455, 1428, 1253, 1186, 1079, 973, 915, 832, 751, 703 cm^−1^. ^1^H NMR (300 MHz, CDCl_3_): δ =7.57 (d, *J* = 6.0 Hz, 1H), 7.25–7.40 (m, 5H), 6.33 (d, *J* = 6.0 Hz, 1H), 5.13 (s, 2H, *CH*_2_Ph), 2.06 (s, 3H, *CH*_3_) ppm. MS (APCI, CH_3_OH) *m*/*z* (%) = 217 (100) [M + H]^+^. C_13_H_12_O_3_ (216.3): calcd. C 72.22, H 5.56; found: C 72.43, H 5.47.

*Synthesis of (3′-aminopropyl)-3-benzyloxy-2-methyl-4-pyridinone* (**2a**). First 3 g 3-Benzyoxy-2-methyl-4-pyrone (13.9 mmol), 3.5 mL 1,3-diaminopropane (42.1 mmol) and 0.5 g NaOH were added in a EtOH–water mixture (20/15) mL, and then stirred at 75 °C for 3 h. After cooling, 2 M HCl was added until the pH = 1, and the reaction mixture was evaporated under vacuum. The residue was washed with acetone and dissolved in 20 mL water. Then the aqueous was basified with 10 M NaOH until pH = 12, and extracted with dichloromethane (5 × 30 mL). The organic solution was evaporated to dryness to obtain the faint yellow oil (3.5 g, 87%). UV-vis (CH_3_OH): λ_max_ = 213, 277 nm. FT-IR (KBr ): ν = 3432 (N-H), 3063, 3030 (C-H, Ar), 2492, 2872 (C-H), 1622(C=O), 1550, 1250, 838, 752, 703 cm^−1^. ^1^H-NMR (300 MHz, CD_3_OD): δ =7.73 (d, *J* = 6.0 Hz, 1H), 7.30–7.42 (m, 5H), 6.47 (d, *J* = 7.4 Hz, 1H), 5.08 (s, 2H, *CH*_2_Ph), 4.03 (t, *J* = 7.5 Hz, 2H), 2.75 (t, *J* = 7.2 Hz, 2H, NH_2_*CH*_2_CH_2_), 2.19 (s, 3H, CH_3_), 1.87 (m, 2H, CH_2_*CH*_2_CH_2_) ppm. MS (APCI) *m*/*z* (%) = 273.0 (100) [M + H]^+^. C_16_H_20_O_2_N_2_ (272.1): calcd. C 70.59, H 7.35, N 10.29; found: C 70.61, H 7.37, N 10.24.

*Synthesis of N,N′-bis(4-(3-benzyloxy-2-methyl-4-pyridinone)propyl)malonamide* (**3a**). First 0.4 g (3*′*-aminopropyl)-3-benzyloxy-2-methyl-4-pyridinone (1.5 mmol) and 2 mL triethylamine were added in 50 mL dichloromethane, then mixture was stirred at 0 °C under N_2_. Then 75 μL malonyl dichloride was diluted with 20 mL dichloromethane and then dropwise added slowly and consecutively keeping the temperature at 0 °C for 8 h. The mixture was purified by chromatography on a silica-gel column with the methanol: chloroform = 1:6 as eluent, the pure product was white powder (0.29 g, 64%). UV-vis (CH_3_OH): λ_max_ = 213, 279 nm. FT-IR (KBr): ν = 3226 (N-H), 3063 (C-H, Ar), 2935, 2879, 1654, 1624, 1559, 1518, 1249, 1249, 1218, 1160, 1036, 975, 833, 750, 703 cm^−1^. ^1^H-NMR (300 MHz, CD_3_OD): δ = 7.72 (d, *J* = 7.2 Hz, 2H), 7.29–7.41 (m, 10H), 6.48 (d, *J* = 7.2 Hz, 2H), 5.07 (s, 4H, C*H*_2_Ph), 4.00 (t, *J* = 7.5 Hz, 4H, NHCH_2_CH_2_C*H*_2_), 3.31 (t, *J* = 6.0 Hz, 4H, NHC*H*_2_CH_2_CH_2_), 3.20 (s, 2H, CO*CH*_2_CO), 2.16 (s, 6H, C*H_3_*), 1.87 (m, 4H, CH_2_C*H*_2_CH_2_) ppm. MS (APCI) *m*/*z* (%) = 613 (100) [M + H]^+^. C_35_H_40_O_6_N_4_ (612): calcd. C 68.63, H 6.54, N 9.15; found: C 68.42, H 6.83, N 9.51.

*Synthesis of N,N′-bis(4-(3-hydroxy-2-methyl-4-pyridinone)propyl)malonamide* (**4a**). First 40 mg 10% Pd/C was added in a solution of *N,N′*-bis(4-(3-benzyloxy-2-methyl-4-pyridinone)propyl)malonamide (0.2 g, 3.7 mmol) in methanol (50 mL), then the mixture was stirred under H_2_ (1 atm) for 4 h at room temperature. After filtration, the solvent was evaporated under reduced pressure and the product was obtained as the white powder (0.13 g, 89%). UV-vis (CH_3_OH): λ_max_ = 279 nm. FT-IR (KBr): ν = 3069, 2930, 1656 (C=OCH_2_), 1627 (PyC=O), 1561, 1509, 1251 (C=ONH_2_), 1035, 832 cm^−1^. ^1^H-NMR (600 MHz, DMSO-*d*_6_): δ =7.64 (d, *J* = 7.2 Hz, 2H), 6.48 (d, *J* = 7.2 Hz, 2H), 4.10 (t, *J* = 7.5 Hz, 4H, NHCH_2_CH_2_C*H*_2_), 3.31 (t, *J* = 6.0 Hz, 4H, NHC*H*_2_CH_2_CH_2_), 3.21 (s, 2H, COC*H*_2_CO), 2.42 (s, 6H, C*H*_3_), 1.96 (m, 4H, CH_2_C*H*_2_CH_2_) ppm. ^13^C-NMR (150 MHz, DMSO-*d*_6_): δ = 169.19, 168.48, 145.95, 137.55, 131.33, 128.01, 51.43, 46.52, 35.98, 30.06, 10.46 ppm. MS (APCI) *m*/*z* (%)= 217 (100) [M + 2H]^+^. C_21_H_28_O_6_N_4_ (432): calcd. C 58.33, H 6.48, N 12.96; found: C 57.97, H 6.51, N 12.85.

*Synthesis of (3′-aminoethyl)-3-benzyloxy-2-methyl-4-pyridinone* (**2b**). First 3 g 3-benzyoxy-2-methyl-4-pyrone (13.9 mmol) and 3.3 mL ethanediamine (49.3 mmol) were added in a EtOH–water mixture (20/15) mL, then 0.5 g NaOH was added and stirred at 70 °C for 4 h. After cooling, 2 M HCl was added until the pH = 1 and the solvent was evaporated. The residue was washed with acetone and dissolved in 20 mL water. The obtained solution was basified with 10 M NaOH until pH ≈ 12 and extracted with dichloromethane (5 × 30 mL). The organic phase was evaporated and obtained the flavescent oil (2.84 g, 74%). UV-vis (CH_3_OH): λ_max_ = 213, 277 nm. FT-IR (KBr): ν = 3433, 1591, 1490, 1384, 1332, 1103, 824, 592 cm^−1^. ^1^H-NMR (300 MHz, CD_3_OD): δ = 7.67 (d, *J* = 7.5 Hz, 1H), 7.30–7.42 (m, 5H), 6.46 (d, *J* = 7.5 Hz, 1H), 5.07 (s, 2H, C*H*_2_Ph), 3.96 (t, *J* = 7.5 Hz, 2H, NH_2_C*H_2_*CH_2_), 2.84 (t, *J* = 7.2 Hz, 2H, NH_2_CH_2_C*H*_2_), 2.17 (s, 3H, C*H*_3_) ppm. MS(APCI) *m*/*z* (%) = 259 (100) [M + H]^+^. C_15_H_18_O_2_N_2_ (258.1): calcd. C 69.77, H 6.98, N 10.85; found: C 70.23, H 7.17, N 10.49.

*Synthesis of* *N,N′-bis(4-(3-benzyloxy-2-methyl-4-pyridinone)ethyl)malonamide* (**3b**). First 0.4 g (3′-aminoethyl)-3-benzyloxy-2-methyl-4-pyridinone (1.5 mmol) and 2 mL triethylamine were added in 50 mL dichloromethane, then mixture was stirred at 0 °C under N_2_. Then 75 μL malonyl dichloride was diluted with 20 mL dichloromethane and then dropwise added slowly and consecutively keeping the temperature at 0 °C for 8 h. The mixture was purified by chromatography on a silica-gel column with the methanol: chloroform = 1:6 as eluent, and the pure product was white powder (0.26 g, 58%). UV-vis (CH_3_OH) λ_max_ = 213, 279 nm. FT-IR (KBr): ν = 2987, 1961, 1676, 1623, 1548, 1507, 1395, 1252, 1164, 1034, 880, 835, 752, 703, 613 cm^−1^. ^1^H-NMR (300 MHz, CD_3_OD): δ = 7.62 (d, *J* = 7.5 Hz, 2H), 7.28–7.44 (m, 10H), 6.44 (d, *J* = 7.5 Hz, 2H), 5.04 (s, 4H, *CH*_2_Ph), 4.07 (t, *J* = 6.0 Hz, 4H, NHCH_2_C*H*_2_), 3.45 (t, *J* = 6.0 Hz, 4H, NHC*H*_2_CH_2_), 3.08 (s, 2H, COC*H*_2_CO), 2.16 (s, 6H, CH_3_). MS (APCI) *m*/*z* (%) = 585 (100) [M + H]^+^. C_33_H_36_N_6_O_4_ (584): calcd. C 67.81, H 6.16, N 9.56; found: C 67.55, H 5.87, N 9.38.

*Synthesis of* *N,N′-bis(4-(3-hydroxy-2-methyl-4-pyridinone)ethyl)malonamide* (**4b**). First 40 mg 10% Pd/C was added in a solution of *N,N′*-bis(4-(3-benzyloxy-2-methyl-4-pyridinone)ethyl)malonamide **3b** (0.2 g, 3.7 mmol) in methanol (50 mL), then the mixture was stirred under H_2_ (1 atm) for 4 h at room temperature. After filtration, the solvent was evaporated under reduced pressure and the product was obtained as the white powder (0.12 g, 83%). UV-vis (CH_3_OH): λ_max_ = 279 nm. FT-IR (KBr): ν = 3429, 3069, 2093, 1654, 1627, 1561, 1509, 1353, 1251, 1035, 831 cm^−1^. ^1^H-NMR (600 MHz, CD_3_OD): δ = 7.62 (d, *J* = 7.5 Hz, 2H), 6.46 (d, *J* = 7.5 Hz, 2H), 4.07 (t, *J* = 6.0 Hz, 4H, NHCH_2_C*H*_2_), 3.45 (t, *J* = 6.0 Hz, 4H, NHC*H*_2_CH_2_), 3.08 (2H, s, COC*H*_2_CO), 2.16(6H, s, C*H_3_*) ppm. MS (APCI) *m*/*z* (%) = 405 (100) [M + H]^+^. C_19_H_24_O_6_N_4_ (404): calcd. C 56.44, H 5.94, N 13.86; found: C 56.38, H 6.25, N 13.74.

*Synthesis of* *N-(4-aminophenyl)-3-benzyloxy-2-methyl-4-pyridinone* (**2c**). First 4.6 g 3-benzyoxy-2-methyl-4-pyrone (21.3 mmol) and 6.9 g *p*-phenylenediamine (63.9 mmol) were added in a mixture solvent of 40 mL EtOH and 20 mL water, then 0.5 g NaOH was added and stirred at 90 °C for 8 h. After cooling, 2 M HCl was added until the pH = 1 and the solvent was evaporated. The residue was washed with acetone and dissolved in 20 mL water. The obtained solution was basified with 10 M NaOH until pH = 12 and extracted with dichloromethane (5 × 30 mL). The organic phase was evaporated under reduced pressure and the residue was dissolved in 10 mL ethanol. Then 200 mL diethyl ether was added to afford the light yellow rude product, which was purified by chromatography on a silica gel column with a mixture of methanol: chloroform = 1:20 as eluent to afford a white powder (6.25 g, 69%). UV-vis (CH_3_OH): λ_max_ = 207, 250, 279 nm. FT-IR(KBr): ν = 3434, 3347 (N-H), 3232 (C-H), 2961 (C-H), 1619 (C=O), 1567, 1511, 1479, 1358, 1281, 1162, 953, 843, 764, 703 cm^−1^. ^1^H-NMR (300 MHz, CDCl_3_): δ = 7.44 (d, *J* = 6.8 Hz, 2H, 7.28–7.36 (m, 3H), 7.23 (d, *J* = 6.8 Hz, 1H), 6.94 (d, *J* = 8.5 Hz, 2H), 6.66 (d, *J* = 8.5 Hz, 2H), 6.41 (d, *J* = 6.8 Hz, 1H), 5.24 (s, 2H, *CH*_2_Ph), 1.82 (t, *J* = 7.5 Hz, 3H, C*H*_3_). MS (ESI) *m*/*z* (%) = 307 (100) [M + H]^+^. C_19_H_18_O_2_N_2_ (306): calcd. C 74.51, H 5.88, N 9.15; found: C 74.32, H 5.92, N 9.13.

*Synthesis of N,N′-bis(4-(3-benzyloxy-2-methyl-4-pyridinone)phenyl)malonamide* (**3c**). First 1.6 g *N*-(4-aminophenyl)-3-benzyloxy-2-methyl-4-pyridinone **2c** was stirred at 0 °C under N_2_. Then 300 μL malonyl dichloride was diluted with 20 mL dichloromethane and then dropwise added slowly and consecutively keeping the temperature at 0 °C for 8 h. The mixture was purified by chromatography on a silica-gel column with the methanol:chloroform = 1:8 as eluent, and the pure product was faint yellow powder (1.5 g, 86%). UV-vis (CH_3_OH): λ_max_ = 208, 251, 281 nm. FT-IR (KBr): ν = 3247, 3189, 3056, 2926, 1697, 1625, 1546, 1508, 1409, 1341, 1287, 1172, 1004, 988, 834, 751, 701 cm^−1^. ^1^H-NMR (300 MHz, CD_3_OD): δ = 7.64 (d, *J* = 8.5 Hz, 4H), 7.22–7.40 (m, 2H), 7.03 (d, *J* = 8.5 Hz, 4H), 6.66 (d, *J* = 7.5 Hz, 4H), 6.42 (d, *J* = 7.5 Hz, 2H), 5.08 (s, 4H, *CH*_2_Ph), 3.45 (s, 2H, COC*H*_2_CO), 1.71 (t, 6H, C*H*_3_) ppm. MS (ESI) *m*/*z* (%) = 681 (100) [M + H]^+^. C_41_H_36_O_6_N_4_ (680): calcd. C 72.35, H 5.29, N 8.24; found: C 72.24, H 5.35, N 8.20.

*Synthesis of N,N′-bis(4-(3-hydroxy-2-methyl-4-pyridinone)phenyl)malonamide* (**4c**). First 50 mg 10% Pd/C was added in a solution of *N,N′*-bis(4-(3-benzyloxy-2-methyl-4-pyridinone)phenyl)malonamide **3c** (0.23 g, 3.38 mmol) in methanol (50 mL), then the mixture was stirred under H_2_ (1 atm) for 4 h at room temperature. After filtration, the solvent was evaporated under reduced pressure and the crude product was obtained as the white powder, which was recrystallized from methanol/diethyl ether (0.15 g, 90%). UV-vis (CH_3_OH): λ_max_ = 209, 251, 292 nm. FT-IR (KBr): ν = 3245 (N-H), 3121, 3063, 2928 (C-H), 1693 (HNC=O), 1626(PyC=O), 1605, 1538, 1505, 1411, 1297, 1243, 1116, 1041, 834 cm^−1^. ^1^H-NMR (600 MHz, CD_3_OD): δ = 7.87 (d, *J* = 7.8 Hz, 4H), 7.64 (d, *J* = 7.2 Hz, 2H), 7.38 (d, *J* = 8.5 Hz, 4H), 6.53 (d, *J* = 7.2 Hz, 2H), 3.63 (s, 2H, COC*H_2_*CO), 2.15 (s, 6H, C*H*_3_) ppm. ^13^C-NMR (150 MHz, CD_3_OD): δ = 165.70, 161.32, 143.03, 139.64, 137.69, 138.63, 136.65, 125.81, 119.98, 109.70, 29.39, 12.41 ppm. MS (ESI) *m*/*z* (%) = 523 (100) [M + Na]^+^. C_27_H_24_O_6_N_4_ (500): calcd. C 64.80; H 4.80, N 11.20; found: C 64.47, H 4.96, N 10.96.

*Synthesis of (3′-hydroxypropyl)-3-benzyloxy-2-methyl-4-pyridinone* (**2d**). First 3 g 3-benzyoxy-2-methyl-4-pyrone (13.9 mmol), 3.5 mL 1,3-propanol amine (45.8 mmol) and 0.5 g NaOH were added in a EtOH–water mixture (20/15) mL, and then stirred at 70 °C for 6 h. After cooling, 2 M HCl was added until the pH = 1 and the reaction solution was evaporated under vacuum. The residue was washed with acetone and dissolved in 20 mL water. The obtained solution was basified with 10 M NaOH until pH = 10 and extracted with dichloromethane (5 × 30 mL). The organic phase was evaporated and obtained the snow white powder (2.9 g, 75%). UV-vis (CH_3_OH): λ_max_ = 216, 274 nm. FT-IR (KBr): ν = 3336, 1632, 1522, 1494, 1419, 1352, 1263, 1159, 1157, 1084, 1035, 960, 842, 754, 706 cm^−1^. ^1^H-NMR (300 MHz, CD_3_OD): δ = 7.62 (d, *J* = 7.4 Hz, 1H), 7.32–7.46 (5H, m), 6.46 (d, *J* = 7.4 Hz, 1H), 5.06 (s, 2H, *CH*_2_Ph), 4.13 (t, *J* = 7.1 Hz, 2H, HOC*H*_2_CH_2_), 4.03 (t, *J* = 7.1 Hz, 2H, HOCH_2_CH_2_C*H*_2_), 2.13 (s, 3H, C*H_3_*), 2.00 (t, *J* = 6.2 Hz, 2H, HOCH_2_C*H*_2_CH_2_) ppm. MS (APCI) *m*/*z* (%) = 274 (100) [M + H]^+^. C_16_H_19_O_3_N (273): calcd. C 70.33,H 6.96, N 5.13; found: C 70.08, H 6.78, N 5.26.

*Synthesis of N,N′-bis(4-(3-benzyloxy-2-methyl-4-pyridinone)hydroxypropyl)malonicester* (**3d**). First 0.5 g (3′-hydroxypropyl)-3-benzyloxy-2-methyl-4-pyridinone (1.83 mmol) and 2 mL triethylamine were added in 50 mL dichloromethane, then mixture was stirred at 0 °C under N_2_. Then 100 μL malonyl dichloride was diluted with 20 mL dichloromethane and then dropwise added slowly and consecutively keeping the temperature at 0 °C for 6 h. The mixture was purified by chromatography on a silica-gel column with the methanol:chloroform =1:8 as eluent, and the pure product was faint yellow oil (0.2 g, 37%). UV-vis (CH_3_OH): λ_max_ = 218, 274 nm. FT-IR (KBr): ν = 2925, 1742, 1647, 1494, 1384, 1263, 1159, 1083, 1035, 842, 753, 706 cm^−1^. ^1^H-NMR (300 MHz, CD_3_OD): δ = 7.18–7.36 (m, 12 H), 6.35 (d, *J* = 7.4 Hz, 2H), 5.08 (s, 4 H, *CH*_2_Ph), 4.06 (d, *J* = 4.5 Hz, 4H, OC*H*_2_CH_2_CH_2_), 3.83 (t, *J* = 6.6 Hz, 2H, OCH_2_CH_2_C*H*_2_), 3.36 (s, 2H, COC*H*_2_CO), 2.02 (s, 6H, C*H*_3_), 1.85–1.95 (m, 4H, OCH_2_C*H*_2_CH_2_) ppm. MS (APCI) *m*/*z* (%) = 308 (100) [M + 2 H]^+^. C_35_H_38_O_8_N_2_ (614): calcd. C 68.40, H 6.19, N 4.55; found: C 68.28, H 6.42, N 4.37.

*Synthesis of N,N′-Bis(4-(3-hydroxy-2-methyl-4-pyridinone)-hydroxyethyl)-malonic ester* (**4d**). First 40 mg 10% Pd/C was added in a solution of *N,N′*-bis(4-(3-hydroxy-2-methyl-4-pyridinone)hydroxyethyl)-malonic ester (0.22 g, 0.34 mmol) in methanol (50 mL), then the mixture was stirred under H_2_ (1 atm) for 6 h at room temperature. After filtration, the solvent was evaporated under reduced pressure and the crude product was obtained as the white powder (0.11 g, 71%). UV-vis (CH_3_OH): λ_max_ = 284 nm. FT-IR (KBr) ν/cm^−1^: 3399, 2965, 2426, 1731, 1632, 1508, 1383, 1255, 1162, 1032, 827. ^1^H-NMR (600 MHz, CD_3_OD): δ = 7.99 (d, *J* = 7.2 Hz, 2H), 6.88 (d, *J* = 7.2 Hz, 2H), 4.39 (t, *J* = 4.5 Hz, 4H, OC*H*_2_CH_2_CH_2_), 4.22 (t, *J* = 6.6 Hz, 4H, OCH_2_CH_2_C*H*_2_), 3.67 (s, 2H, COC*H_2_*CO), 2.17 (s, 6H, CH_3_), 2.00–2.20 (m, 4H, OCH_2_C*H_2_*CH_2_); MS (APCI) *m*/*z* (%) = 435 (100) [M + H]^+^. C_21_H_26_O_8_N_2_ (434): calcd. C 58.06, H 5.99, N 6.45; found: C 58.13, H 6.07, N 6.21.

*Synthesis of (3′-Hydroxyethyl)-3-benzyloxy-2-methyl-4-pyridinone* (**2e**). First 4.3 g 3-benzyoxy-2-methyl-4-pyrone (19.9 mmol), 3.3 mL 2-aminoethanol (49.3 mmol) and 0.5 g NaOH were added in a mixture solvent of 20 mL EtOH and 15 mL water mixture, and then stirred at 70 °C for 6 h. After cooling, 2 M HCl was added until the pH = 1 and the reaction solution was evaporated under vacuum. The residue was washed with acetone and dissolved in 20 mL water. The obtained solution was basified with 10 M NaOH until pH = 10 and extracted with dichloromethane (5 × 30 mL). The organic phase was evaporated and obtained the snow white powder (4.6 g, 89%). UV-vis (CH_3_OH): λ_max_ = 215, 270 nm. FT-IR (KBr): ν = 3335, 1636 (C=O), 1523, 1492, 1342, 1295, 1271, 1157, 1079, 1075, 1032, 969, 831, 768, 708 cm^−1^. ^1^H-NMR (300 MHz, CD_3_OD): δ = 8.32 (1 H, d, *J* = 7.2 Hz), 7.36–7.44 (m, 5H), 7.23 (d, *J* = 7.2 Hz, 1H), 5.17 (s, 2H, C*H*_2_Ph), 4.47 (t, *J* = 7.5 Hz, 2H, HO*CH_2_*CH_2_), 3.89 (t, *J* = 7.5 Hz, 2H, HOCH_2_C*H*_2_), 2.51 (s, 3H, C*H*_3_) ppm. MS (APCI) *m*/*z* (%)= 260 (100) [M + H]^+^. C_15_H_17_O_3_N (259): calcd. C 69.50, H 6.56, N 5.41; found: C 69.84, H 6.72, N 5.38.

*Synthesis of N,N′-bis(4-(3-benzyloxy-2-methyl-4-pyridinone)-Hydroxyethyl)-malonic ester* (**3e**). First 0.5 g (3′-hydroxyethyl)-3-benzyloxy-2-methyl-4-pyridinone (1.9 mmol) and 2 mL triethylamine were added in 50 mL dichloromethane, then mixture was stirred at 0 °C under N_2_. Then 100 μL malonyl dichloride was diluted with 20 mL dichloromethane and then dropwise added slowly and consecutively keeping the temperature at 0 °C for 6 h. The mixture was purified by chromatography on a silica-gel column with the methanol: chloroform=1:8 as eluent, and the pure product was faint yellow oil (0.12 g, 35%). UV/vis (CH_3_OH): λ_max_ *=* 220, 273 nm. FT-IR (KBr): ν = 2921, 2851, 1748, 1625, 1561, 1512, 1251, 1230, 1108, 1064, 825, 737, 701 cm^−1^. ^1^H-NMR (400 MHz, CD_3_OD): δ = 8.32 (d, *J* = 7.2 Hz, 2H), 7.36–7.44 (m, 10H), 7.26 (d, *J* = 7.2 Hz, 2H), 5.17 (s, 4H, C*H*_2_Ph), 4.47 (t, *J* = 7.8 Hz, 2H, OC*H*_2_CH_2_), 3.89 (t, *J* = 7.8 Hz, 2H, OCH_2_C*H*_2_), 3.69 (s, 2H, COC*H*_2_CO), 2.51(s, 3H, C*H_3_*). MS (APCI) *m*/*z* (%) = 587 (100) [M + H]^+^. C_33_H_34_O_8_N_2_ (586): calcd. C 67.58,; H 5.80, N 4.78; found: C 67.66, H 6.01, N 4.64.

*Synthesis of N,N′-Bis(4-(3-hydroxy-2-methyl-4-pyridinone)-hydroxyethyl)-malonic ester* (**4e**). First 40 mg 10% Pd/C was added in a solution of *N*,*N′*-bis(4-(3-hydroxy-2-methyl-4-pyridinone)-hydroxyethyl)-malonic ester (0.19 g, 0.34 mmol) in methanol (50 mL), then the mixture was stirred under H_2_ (1 atm) for 6 h at room temperature. After filtration, the solvent was evaporated under reduced pressure and the crude product was obtained as the white powder, which was recrystallized from methanol/diethyl ether (0.10 g, 76%). UV-vis (CH_3_OH) λ_max_ = 281 nm. FT-IR (KBr): ν = 3367, 2963, 1736, 1628, 1567, 1508, 1460, 1384, 1352, 1251, 1150, 1110, 1026, 831, 599 cm^−1^. ^1^H-NMR (600 MHz, CD_3_OD): δ = 8.31 (d, *J* = 7.2 Hz, 2H), 7.25 (d, *J* = 7.2 Hz, 2H), 4.38 (t, *J* = 7.8 Hz, 2H, OC*H*_2_CH_2_), 3.91 (t, *J* = 7.8 Hz, 2H, OCH_2_C*H*_2_), 3.67 (s, 2H, CO*CH_2_*CO), 2.49 (s, 3H, CH_3_). MS (APCI) *m*/*z* (%) = 407 (100) [M + H]^+^. C_19_H_22_O_8_N_2_ (406): calcd. C 56.16, H 5.42, N 6.90; found: C 56.32, H 5.63, N 7.02.

### 3.3. Metal Complexation Solutions

In all the complexation studies in aqueous solution, the water was distilled three times and the atmospheric CO_2_ was excluded from the system with a purging steam of N_2_ under 80 °C. The UO_2_(NO_3_)_2_·6H_2_O is analytical grade, the buffered solution were NaAc/HAc (pH = 5.5) and Tris-HCl (pH = 7.4, 9.0). All the ligands were synthesized and dissolved into the water, except the solvent of the ligand **4c** was DMSO:H_2_O = 1:9 to ensure all the ligands was dissolved. The ligands concentration (C_L_) was 2.0 × 10^−3^ mol/L and the UO_2_^2+^ (C_M_) concentration was 2.0 × 10^−3^ mol/L, then added the solutions into the 10 mL comparison tubes by different volume, the total concentration (C_L_ + C_M_) was 2.0 × 10^−3^ mol/L for all complexation samples.

## 4. Conclusions

In summary, five new ligands of bis(3-hydroxy-4-pyridinone) tetradentate ligands were synthesized and characterized. The stability constants determined in this study provide evidence for the extremely high affinities of the ligands for UO_2_^2+^ complexes. The evident difference in logK_cond_, compared with the similar structural ligands, emphasized the superior affinity for UO_2_^2+^ of the **4b** ligand. At pH 7.4, the **4b** ligand shows a high constant of 22.7, confirming this compound as an effective chelator for UO_2_^2+^.

## Figures and Tables

**Table 1:** Summary of the logK_cond_ of ligand-UO_2_^2+^ (pH = 7.4 ± 0.1).

L	logK_cond_U-L/(pH = 7.4)
**4a**	21.7
**4b**	22.7
**4c**	18.6
**4d**	21.4
**4e**	22.2

## References

[1] Hamilton J.G. (1948). The metabolic properties of the fission products and actinide elements. Rev. Mod. Phys..

[2] Brugge D.J., Lemos L.D., Oldmixon B. (2005). Exposure pathways and health effects associated with chemical and radiological toxicity of natural uranium: A review. Rev. Environ. Health.

[3] Morss L.R., Edelstein N.M., Fuger J., Katz J.J. (2006). The Chemistry of the Actinide and Transactinide Elements.

[4] Guzmán L., Durán-Lara E.F., Donoso W., Nachtigall F.M., Santos L.S. (2015). *In Vivo* Nanodetoxication for acute uranium exposure. Molecules.

[5] Liu G., Bruenger F.W., Miller S.C., Afri A.M. (1998). Molecular structure and biological and pharmacological properties of 3-hydroxy-2-methyl-1-(β-d-ribofuranosyl or pyranosyl)-4-pyridinone: Potential iron overload drugs for oral administration. Bio. Med. Chem. Lett..

[6] Santos M.A., Gil M., Marques S., Gano L., Cantinho G.J. (2002). N-Carboxyalkyl derivatives of 3-hydroxy-4-pyridinones: Synthesis, complexation with Fe(III), Al(III) and Ga(III) and *in vivo* evaluation. J. Inorg. Biochem..

[7] Santos M.A., Gama S., Gano L., Farkas E. (2005). Bis(3-hydroxy-4-pyridinone)-EDTA derivative as a potential therapeutic Al-chelating agent. Synthesis, solution studies and biological assays. J. Inorg. Biochem..

[8] Santos M.A., Grazina R., Buglyo P., Gama S., Farkas E. (2002). A new bipodal carboxy-bis (hydroxypyridinonate) ligand. Synthesis and complexation with copper(II), nicker(II) and zinc(II) in aqueous solution. Polyhedron.

[9] Leite A., Silva A.M.G., Nunes A., Andrade M., Sousa C., Silva L.C., Gameiro P., Castro B.D., Rangel M. (2011). Novel tetradentate chelators derived from 3-hydroxy-4-pyridinone units: synthesis, characterization and aqueous solution properties. Tetrahedron.

[10] Zhou T., Liu Z.D., Neubert H., Kong X.L., Ma Y.M., Hider R.C. (2005). High affinity iron(III) scavenging by a novel hexadentate 3-hydroxypyridin-4-one-based dendrimer: Synthesis and characterization. Bioorg. Med. Chem. Lett..

[11] Grazina R., Gano L., Šebestík J., Santos M.A. (2009). New tripodal hydroxypyridinone based chelating agents for Fe(III), Al(III) and Ga(III): Synthesis, physico-chemical properties and bioevaluation. J. Inorg. Biochem..

[12] Salaheldin A.M., Al-Sheikh M.A. (2010). β-Enamino esters in heterocyclic synthesis: Synthesis of pyrazolone and pyridinone derivatives. Molecules.

[13] Xu B., Kong X.L., Zhou T., Qiu D.H., Chen Y.L., Li M.S. (2011). Synthesis, iron(III)-binding affinity and *in vitro* evaluation of 3-hydroxypyridin-4-one hexadentate ligands as potential antimicrobial agents. Bioorg. Med. Chem. Lett..

[14] Santos M.A., Marques S.M., Chaves S. (2012). Hydroxypyridinones as privileged chelating structures for the design of medicinal drugs. Coord. Chem. Rev..

[15] Crisponi G., Remeli M. (2008). Iron chleating agent for the treatment of iron overload. Coord. Chem. Rev..

[16] Santos M.A. (2008). Recent development on 3-hydroxy-4-pyridinones with respect to their clinical applications Mono and combined ligand approaches. Coord. Chem. Rev..

[17] Gama S., Gil M., Gano L., Farkas E., Santos M.A. (2009). Combined chelation of bi-functional bis-hydroxypiridinone and mono-hydroxypiridinone: Synthesis, solution and *in vivo* evaluation. J. Inorg. Biochem..

[18] Santos M.A., Gama S., Gano L., Cantinho G., Frakas E. (2004). A new bis(3-hydroxy-4-pyridinone)-IDA derrivatives as a potential therapeutic chelating agent. Synthesis,metal-complexation and biological assays. Dalton Trans..

[19] Zhou T., Neubert H., Liu D.Y., Liu Z.D., Ma Y.M., Kong X.L., Luo W. (2006). Iron dendrimers: A novel approach for the treatment of Haemochromatosis. J. Med. Chem..

[20] Leydier A., Lecerclé D., Rostaing S.P., Reguillon A.F., Taran F., Lemaire M. (2011). Sequestering agent for uranyl chelation: New binaphtyl ligands. Tetrahedron Lett..

[21] Hider R.C., Roy S., Ma Y.M., Kong X.L., Preston J. (2011). The potential application of iron chelators for the treatment of neurodegenerative disease. Metallomics.

[22] Santos M.A., Grazina R., Neto A.Q., Cantinho G., Gano L., Patrício L. (2000). Synthesis, chelating poperties towards gallium and biological evaluation of two *N*-substituted 3-hydroxy-4-pyridinones. J. Inorg. Biochem..

[23] Leydier A., Lecerclé D., Rostaing S.P., Réguillona A.F., Taran F., Lemairea M. (2008). Sequestering agents for uranyl chelation: New calixarene ligands. Tetrahedron.

[24] Hoehne M.S., Deblonde G.J.P., Abergel R.J. (2013). Solution thermodynamic evaluation of hydroxypyridinonate chelators 3,4,3-LI(1,2-HOPO) and 5-LIO(Me-3,2-HOPO) for UO_2_(VI) and Th(IV) decorporation. Radiochim. Acta.

[25] Szigethy G., Raymond K.N. (2011). Hexadentate Terephthalamide (bis-hydroxypyridinone) ligands for uranyl chelation: Structural and thermodynamic consequences of ligand variation. J. Am. Chem. Soc..

[26] Chaves S., Capelo A., Areias L., Marpues S.M., Gano L., Esteves M.A., Santos M.A. (2013). A novel tripodal tris-hydroxypyrimidinone sequestering agent for trivalent hard metal ions: Synthesis, complexation and *in vivo* studies. Dalton Trans..

[27] Bartholomä M.D. (2012). Recent developments in the design of bifunctional chelators for metal-based radiopharmaceuticals used in positron emission tomography. Inorg. Chim. Acta.

[28] Chaves S., Marques S.M., André M.F. (2010). New tris(hydroxypyridinones) as iron and aluminium sequestering agents: Synthesis, complexation and *in vivo* studies. Chem. Eur. J..

[29] Xu J.D., Raymond K.N. (1999). Uranyl sequestering agents: Correlation of properties and efficacy with structure for UO_2_^2+^ complexes of linear tetradentate 1-methyl-3-hydroxy-2(1*H*)-pyridinone ligands. Inorg. Chem..

[30] Domingo J.L., Ortega A., Llobet J.M., Paternain J.L., Corbella J. (1989). The effects of uranium on reproduction, gestation, and postnatal survival in mice. Res. Commun. Pathol. Pharmacol..

[31] Stradling G.N., Gray S.A., Moody J.C., Ellender M. (1991). Efficacy of Tiron for Enhancing the Excretion of Uranium from the Rat. Hum. Exp. Toxicol..

